# Associations of Type 2 Diabetes with Common Variants in *PPARD* and the Modifying Effect of Vitamin D among Middle-Aged and Elderly Chinese

**DOI:** 10.1371/journal.pone.0034895

**Published:** 2012-04-11

**Authors:** Ling Lu, Ying Wu, Qibin Qi, Chen Liu, Wei Gan, Jingwen Zhu, Huaixing Li, Xu Lin

**Affiliations:** Key Laboratory of Nutrition and Metabolism, Institute for Nutritional Sciences, Shanghai Institutes for Biological Sciences, Chinese Academy of Sciences and Graduate School of the Chinese Academy of Sciences, Shanghai, China; Universita Magna-Graecia di Catanzaro, Italy

## Abstract

**Background:**

Previous studies have identified that variants in peroxisome proliferator-activated receptor PPAR-δ (*PPARD*), a target gene of vitamin D, were significantly associated with fasting glucose and insulin sensitivity in European populations. This current study sought to determine (1) whether the genetic associations of PPARD variants with type 2 diabetes and its related traits could be replicated in Chinese Han population, and (2) whether the associations would be modified by the effect of vitamin D status.

**Methods and Findings:**

We genotyped 9 tag single nucleotide polymorphisms (SNPs) that cover the gene of *PPARD* (rs2267664, rs6902123, rs3798343, rs2267665, rs2267668, rs2016520, rs2299869, rs1053049, and rs9658056) and tested their associations with type 2 diabetes risk and its related traits, including fasting glucose, insulin and HbA1c in 3,210 Chinese Hans. Among the 9 *PPARD* tag SNPs, rs6902123 was significantly associated with risk of type 2 diabetes (odds ratio 1.75 [95%CI 1.22–2.53]; *P* = 0.0025) and combined type 2 diabetes and impaired fasting glucose (IFG) (odds ratio 1.47 [95%CI 1.12–1.92]; *P* = 0.0054). The minor C allele of rs6902123 was associated with increased levels of fasting glucose (*P* = 0.0316) and HbA1c (*P* = 0.0180). In addition, we observed that vitamin D modified the effect of rs6902123 on HbA1c (*P* for interaction = 0.0347).

**Conclusions/Significance:**

Our findings demonstrate that common variants in *PPARD* contribute to the risk of type 2 diabetes in Chinese Hans, and provided suggestive evidence of interaction between 25(OH)D levels and *PPARD*-rs6902123 on HbA1c.

## Introduction

Peroxisome proliferator-activated receptor (PPAR)-δ, a member of PPAR family, is widely expressed in a variety of tissues, and regulated by several environmental factors like vitamin D [1] and physical activity [2], which are influencing factors for type 2 diabetes [3], [4]. Evidence from *in vitro* study indicated that PPAR-δ-specific agonist promoted glucose uptake in cultured primary human skeletal myotubes [5]. Animal studies found that PPAR-δ knockout mice showed glucose intolerance on normal chow [6], and were prone to obesity on high-fat diet [7]. Meanwhile, treatment with PPAR-δ-specific agonist (GW501516) could enhanced β-oxidation, decreased free fatty acid, and improved insulin sensitivity in *db/db* mice [6] and moderately obese men [8]. Hence, PPAR-δ is recognized as a target for treatment of metabolic syndromes and type 2 diabetes [9], [10].

The effects of *PPARD* variants on type 2 diabetes and the metabolic related traits has been widely investigated, however, the results were inconsistent. Previous candidate gene association studies in Europeans have reported that common variants in *PPARD* were associated with fasting glucose [11], insulin resistance [11], [12], BMI [13], LDL-cholesterol [14], HDL-cholesterol [13], [15],and risk of conversion from impaired glucose tolerance (IGT) to type 2 diabetes [16]. In Asian population, only two case-control studies were conducted in Korea [17] and China [18], which found that variants in *PPARD* (rs2016520, rs9658173, rs1053049 and c.2806 C>G) were associated with fasting glucose and/or BMI in Korean [17], and the C allele of rs2016520 was associated with higher fasting glucose and lower insulin sensitivity in Chinese [18]. These findings suggested a contribution of *PPARD* to the risk of type 2 diabetes and related metabolic traits.

It is well-established that type 2 diabetes is a common complex disease influenced by both genetic factors and environmental factors [19]. In order to completely understand the role of *PPARD* in etiology of type 2 diabetes, investigation on the interactions between environmental factors and common genetic variants in *PPARD* is required. Vitamin D, which was associated with glucose homeostasis in our previous study [20], was shown to be a regulator of *PPARD* expression [1], and the vitamin D receptor response elements was found in the promoter of *PPARD* [1]. Hence, Vitamin D status might influence the effect of variants in *PPARD* on risk of type 2 diabetes and related traits. Despite of the important role of vitamin D in glucose homeostasis [4], [20], previous studies did not assess the potential gene-nutrients interaction between the variants in *PPARD* and vitamin D status.

Therefore, the primary aim of this study is to investigate the effect of *PPARD* SNPs on the risk of having type 2 diabetes and its related phenotype in a relative large population-based Han Chinese sample. Given the regulative effect of vitamin D on PPAR-δ expression [1], we also sought to examine the potential modifying effects of plasma 25-hydoxyvitamin D (25[OH]D) levels, the indicator of vitamin D status, on the associations between *PPARD* common variants and type 2 diabetes as well as its related traits.

## Materials and Methods

### Study Population

The present study consisted of 3210 unrelated Chinese Hans (1423 men and 1787 women) aged 50–70 years from Beijing and Shanghai, which is part of the Nutrition and Health of Aging Population in China (NHAPC) projects, a population-based cohort. The details of the study design and protocols have been described previously [21]. In brief, in a home interview, information about age, sex, geographical region (Beijing/Shanghai), health status, medication use and physical activity was collected by a standardized questionnaire. After the home interview, all participants attended a clinical examination, including anthropometric measurements and overnight blood sample collection. Height, weight, blood pressure, fasting glucose, insulin, HbA1c and 25(OH)D were measured following standard protocols which were described previously [20], [21]. BMI was calculated as weight in kilograms divided by the square of height in meters. Homeostasis model assessment of insulin resistance (HOMA-R) and of beta cell function (HOMA-B) was estimated using updated homeostasis model assessment methods [22]. The study was approved by the institutional review board of the Institute for Nutritional Sciences, and all participants provided informed consent. The phenotypic characteristics of the population are shown in Table 1.

**Table 1:** Characteristics of study population.

Characteristics	All	Beijing	Shanghai
n	3210	1574	1636
Male (%)	1423 (44.3)	711 (45.2)	712 (43.5)
Age (years)	58.6±6.0	58.3±5.9	58.9±6.0
BMI (kg/m^2^)	24.4±3.6	25.2±3.7	23.6±3.3
Fasting glucose (mmol/l)	5.84±1.74	6.16±1.96	5.53±1.42
HbA1c (%)	5.99±1.10	6.08±1.22	5.90±0.96
Fasting insulin (pmol/l)	82.2 (59.4–112.2)	80.7 (57.6–110.4)	84.0 (61.8–114.0)
HOMA-B (%)	110.3±47.0	100.1±44.9	120.0±46.9
HOMA-IR	1.57 (1.15–2.12)	1.56 (1.12–2.11)	1.57 (1.18–2.13)
IFG (%)	878 (27.4)	579 (36.8)	299 (18.3)
Type 2 diabetes (%)	424 (13.2)	272 (17.3)	152 (9.3)
25(OH)D (nmol/L)	41.0 (31.3–54.3)	35.8 (27.5–45.9)	47.6 (36.6–60.4)

Unless otherwise indicated, data are means ± SD, median (interquartile range) or n (%).

### Definition of Type 2 diabetes

Type 2 diabetes was defined by either 1999 World Health Organization criteria [23] or previously diagnosed type 2 diabetes. Normal fasting glucose (NFG) and impaired fasting glucose (IFG) were defined as fasting glucose <5.6 mmol/l (100 mg/dl) and 5.6 mmol/l to 7.0 mmol/l (126 mg/dl), respectively. Among the participants, 424 had type 2 diabetes (267 previously diagnosed, 157 screen-detected and treatment-naïve), 878 had impaired fasting glucose (IFG) (all screen-detected and treatment-naive), and 1908 had normal fasting glucose (NFG).

### Genotyping

DNA extraction from peripheral blood leucocytes was performed by a salting out procedure (http://www.protocol-online.org/prot/Detailed/3171.html, accessed 1 January 2009). Haploview software (available at http://www.broadinstitute.org/haploview/haploview) were applied to select the nine *PPARD* variants (rs2267664, rs6902123, rs3798343, rs2267665, rs2267668, rs2016520, rs2299869, rs1053049 and rs9658056), which captured all SNPs with minor allele frequencies (MAFs)≥0.05 based on HapMap-CHB database (Rel24/phase II Nov08) in the gene of *PPARD* and 5 kb of its upstream and downstream regions (Table S1). These nine SNPs were genotyped using the GenomeLab SNPstream system (Beckman Coulter, Fullerton, CA, USA). The genotyping success rate was >96%, and the concordance rate was >99% based on 12% duplicated samples (n = 384). All the nine SNPs genotyped in our study were in Hardy-Weinberg equilibrium (*P*>0.01). The SNP rs9658056 was not included in statistical analyses due to its very low frequency (MAF = 0.003) in our samples. Genotypic distributions were similar in Beijing and Shanghai subpopulations (*P*>0.05), except rs3798343, rs2299869 and rs2267664 (*P* = 0.0001, 0.0221 and <0.0001 for rs3798343, rs2299869 and rs2267664, respectively).

### Statistical analyses

Insulin and HOMA-R were log-transformed to approach normal distributions. Due to the differences of phenotypic characteristics between individuals from Beijing and Shanghai, association analyses for all SNPs were performed in individuals from Beijing and Shanghai separately. Subsequently, we applied fixed-effect meta-analysis to combine the summary statistics of the associations from the two subpopulations. A logistic regression model was used to evaluate the association of each SNP with type 2 diabetes and combined IFG/type 2 diabetes under the additive or dominant models (if counts of minor allele homozygote <10). For quantitative traits analyses, participants with known diabetes or receiving glucose-lowering treatment (n = 267) were excluded. Generalized linear regression was applied to quantitative traits analyses under additive or dominant model. The potential modifying effects of 25(OH)D levels on genetic associations with type 2 diabetes or its related traits were evaluated by introducing a gene x nutrient interactive term into the logistic or linear regression models. All association analyses were adjusted for age, sex, and BMI. Quanto program (http://hydra.usc.edu/gxe/) was used for power calculation. All reported *P* values are nominal and two-sided. The statistical analyses were performed using SAS version 9.1 (SAS Institute, Cary, NC, USA).

## Results

All effect-allele frequencies observed in this study were comparable to those in HapMap-CHB sample. Three of the eight SNPs (rs2299869, rs2267664 and rs3798343) have higher effect-allele frequencies in Chinese Hans than those in HapMap-CEU population, while allele-frequencies were similar for the remaining five SNPs (Table 2 and Table S2). Linkage disequilibrium (LD) pattern among these SNPs was shown in Figure S1.

**Table 2:** Associations of rs6902123 in *PPARD* with type 2 diabetes and combined phenotype of type 2 diabetes and impaired fasting glucose in Chinese Hans.

SNP ID	Effect/Non-effect allele	*P*_HW_	HapMap-CHB	HapMap-CEU	T2DM vs Normal				(T2DM+IFG) vs Normal			
					Effect-allele frequency		OR (95%CI)	*P* a	Effect-allele frequency		OR (95%CI)	*P* a
					Casen = 424	Controln = 1908			Casen = 1302	Controln = 1908		
rs6902123^b^	[C/T]		0.06	0.08								
Beijing		0.4291			0.06	0.04	1.50 (0.92–2.46)	0.1063	0.05	0.04	1.30 (0.89–1.90)	0.1692
Shanghai		0.3975			0.08	0.03	2.12 (1.23–3.64)	0.0065	0.06	0.03	1.66 (1.13–2.44)	0.0102
Combined^c^							1.75 (1.22–2.53)	0.0025			1.47 (1.12–1.92)	0.0054
*P* for heterogeneity								0.3612				0.3752

a The *P* values were adjusted for age, sex, and BMI.

b Dominant model was applied.

c Fixed-effect model was used in the meta-analysis.

We investigated the associations of variants in *PPARD* with the risks of type 2 diabetes in a Chinese Han population by case-control analyses. Among the eight variants we studied, the SNP rs6902123 showed significant association with type 2 diabetes (OR 1.75, 95%CI [1.22–2.53], *P* = 0.0025) and combined IFG/type 2 diabetes (OR 1.47, 95%CI [1.12–1.92], *P* = 0.0054) under a dominant model (Table 2). The associations remained significant after Bonferroni corrections (*P*<0.0063, 0.05/8 tests). However, no further association with type 2 diabetes or combined IFG/type 2 diabetes were detected for the rest PPARD variants in our study (Table S2).

In quantitative traits analyses, we identified that the minor allele of rs6902123 was significantly associated with higher levels of fasting glucose (*P* = 0.0316) and HbA1c (*P* = 0.0180) (Table 3). No evidence of associations with other PPARD SNPs was observed in this Chinese Han population (Table S3). In addition, no significant association was observed after Bonferroni correction.

**Table 3:** Associations with type 2 diabetes related quantitative traits in 2,943 Chinese Hans.

SNP ID	Glucose (mmol/L)^a^		HbA1c (%)^a^		Insulin (mmol/L)^a^ ^b^		HOMA-B (%)^a^		HOMA-IR (%)^a^ ^b^	
	*β* (SE)	*P*	*β* (SE)	*P*	*β* (SE)	*P*	*β* (SE)	*P*	*β* (SE)	*P*
rs6902123^c^										
Beijing	0.301 (0.138)	0.0296	0.241 (0.090)	0.0073	0.002 (0.049)	0.9740	−5.883 (4.018)	0.1433	−0.015 (0.046)	0.7416
Shanghai	0.107 (0.099)	0.2773	0.064 (0.067)	0.3362	0.030 (0.046)	0.5128	0.218 (4.318)	0.9598	0.032 (0.045)	0.4802
Combined^d^	0.173 (0.080)	0.0316	0.127 (0.054)	0.0180	0.017 (0.034)	0.6147	−3.050 (2.940)	0.2995	0.009 (0.032)	0.7793
*P* for heterogeneity		0.2533		0.1147		0.6770		0.301		0.4652

a The *P* values were adjusted for age, sex and BMI.

b Log-transformed before analyses.

c Dominant model was applied.

d Fixed-effect model was used in the meta-analysis.

**Figure 1:**
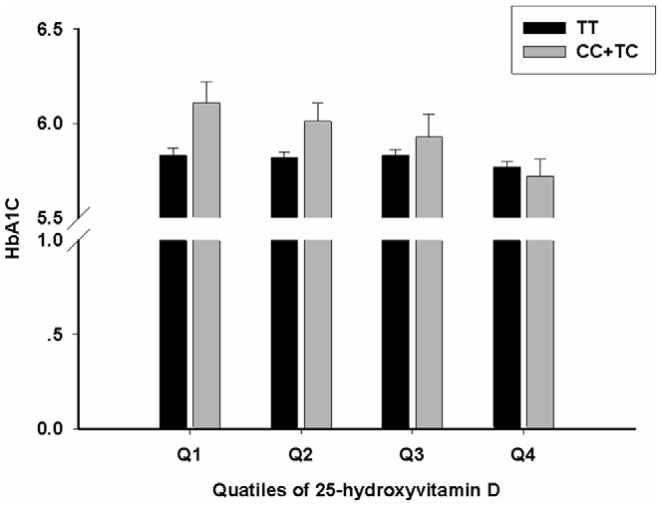
Interaction between *PPARD*-rs6902123 and plasma 25-hydroxyvitamin D levels on HbA1c. The means (SE) of HbA1c by *PPARD*-rs6902123 genotypes according to quartiles of plasma 25-hydroxyvitamin D concentrations were calculated in 2943 subjects, with adjustment for age, sex, geographic region (Beijing and Shanghai) and BMI (P for interaction = 0.0347). Participants with known diabetes or using glucose-lowering treatment were excluded. C allele carriers (black) had significant higher HbA1c concentrations than TT carriers (gray) (P = 0.0172) in the lowest group of 25-hydroxyvitamin D, but not in the other groups (P≥0.0814).

Next, an exploratory study was conducted to assess the interactive effects of *PPARD*-rs6902123 and 25(OH)D levels on type 2 diabetes, combined IFG/type 2 diabetes, and their related traits. However, potential interaction was only detected between rs6902123 and quartiles of 25(OH)D for HbA1c levels (*P* for interaction = 0.0347), but not for other phenotypes in Chinese Hans (Figure 1). In stratified analysis, the C allele carriers of rs6902123 were only found to have significantly increased HbA1c than the TT genotype carriers in the lowest quartile of 25(OH)D (*P* = 0.0172), suggesting that increasing 25(OH)D would attenuated the risk effect of C allele of rs6902123 on HbA1c level.

## Discussion

In this study of Chinese Han population, we systematically investigated the SNPs in *PPARD* by a gene-wide tagging approach, instead of testing only a few SNPs. The *PPARD*-rs6902123 C allele exhibited significant associations with increased risk of type 2 diabetes, combined IFG/type 2 diabetes, higher fasting glucose and HbA1c. Further analyses suggested an interaction between rs6902123 and 25(OH)D on HbA1c.

Previous studies provided controversial results on associations between *PPARD*-rs6902123 and type 2 diabetes and its related traits. In the STOP-NIDDM Trial, 2.47-fold increased risk of conversion from impaired glucose tolerance to type 2 diabetes was observed in female carriers of the rs6902123 C allele [16], which was in line with our results. However, one study in white population reported that common variants in *PPARD*, including rs6902123, did not significantly affect the risk of metabolic disease, except that a modest significant association was observed between the rare alleles of rs947001, rs6457816 and rs9658119 and increased fasting glucose [11]. This discrepancy may be due to the different LD pattern between Chinese Hans and European. The variant rs6902123 is in complete linkage disequilibrium (LD r^2^ = 1) with SNPs of rs947001 and rs6457816 in Chinese Hans, but modestly correlated with the two SNPs in Europeans (LD r^2^ = 0.54 and 0.89 for rs947001 and rs6457816, respectively). Since there is no prior evidence of the potential functional role of the associated variant rs6902123, whether it is a causal variant or is in LD with the causal variant need to be determined in further studies using fine-mapping or re-sequencing strategies, as well as the functional assays.

The underlying mechanisms responsible for the association between *PPARD*-rs6902123 and type 2 diabetes remain to be elucidated. *PPARD* knock out mice are glucose intolerant, whereas treatment of *db/db* mice with PPAR-δ-specific agonist decreased hepatic glucose production, increased insulin stimulated-glucose disposal rate, lower free fatty acid levels, and subsequently improved insulin sensitivity [6]. Nevertheless, we failed to provide evidence for associations between *PPARD*-rs6902123 and HOMA-R. Absence of this association in our study might partially be attributable to insufficient power, since we only have 40% power to detect a beta value of 0.05 for log-transformed HOMA-R at *P*<0.05. Moreover, evidence from *in vitro* study indicated PPAR-δ-specific agonist promoted glucose uptake in cultured primary human skeletal myotubes by increasing AMPK expression and phosphorylation in the absence of insulin, suggesting PPAR-δ-specific agonist directly improved glucose uptake through an insulin-independent mechanism and enhanced subsequent insulin stimulation [5].

For *PPARD*-rs2016520 variant, we only found a marginal association with combined IFG/type 2 diabetes, but not with the risk of type 2 diabetes and related traits. In line with our results, rs2016520 was also not associated with the risk of type 2 diabetes in a Chinese case-control study, in which the subjects were recruited from the out-patient clinic [18]. However, they found that rs2016520 C allele was associated with higher fasting glucose and impaired insulin sensitivity in both normal glucose tolerant and diabetic subjects [18]. This discrepancy may be due to the different sampling method and population structure between the two studies. Our study was a population-based study among non-institutionalized Chinese people in Beijing and Shanghai. This design has the advantage of allowing estimation of the genetic effect at the population level among Chinese Han middle-aged people. In addition, the possibility of false-negative results caused by insufficient power could not be completely excluded. Assuming an additive model and a minor allele frequency of 26%, we had less than 50% power to detect the previously reported beta value for fasting glucose at *P*<0.05 [18]. Meta-analysis or studies with larger sample sizes will be required to draw definitive conclusions.

No evidence of associations with other *PPARD* SNPs was observed in this Chinese Han population. This is very likely attributable to the weak linkage disequilibrium between these variants and the causal variants. However, we could not rule out the possibility of insufficient power. Assuming an additive model and a minor allele frequency of 20%, we had 43% and 79% power to detect the effect of a SNP with odds ratio 1.3 at P<0.05 for risk of type 2 diabetes and combined IFG/type 2 diabetes, respectively, in Shanghai subpopulation, and 56% and 85% power for risk of type 2 diabetes and combined IFG/type 2 diabetes, respectively, in Beijing subpopulation. In addition, we only have 59% and 60% power to identify a SNP which can explain 0.3% variance of the quantitative traits in Beijing and Shanghai subpopulation, respectively. Thus, further larger studies are warranted.

Given the regulative effect of vitamin D on the expression of *PPARD* [1], we also assessed the interaction between *PPARD*-rs6902123 and 25(OH)D levels, the accepted indicator of vitamin D status. We found a significant modifying effect of 25(OH)D levels on the association of *PPARD*-rs6902123 with HbA1c. The *PPARD*-rs6902123 C allele was significantly associated with higher HbA1c level only in the lowest group of 25(OH)D levels. The mechanism underlying this interaction between 25(OH)D levels and rs6902123 is unclear. Vitamin D is a steroid, which play an important role in insulin secretion and glucose uptake through directly modulating gene expression via vitamin D receptors (VDRs) as well as through regulating calcium flux [24]. In our previous study, we found that 25(OH)D, generally accepted indicator of vitamin D status, was associated with fasting glucose, HbA1c and insulin resistance in Chinese middle-aged population [20]. In addition, a VDR response element was found in the promoter of *PPARD* [1]. Therefore, we assume that the higher level of vitamin D status may alleviate the negative effect of *PPARD*-rs6902123 C allele on HbA1c by regulating the expression of *PPARD*. Although, the interaction was no longer significant after Bonferroni correction, it might result from the relatively smaller sample size in our study for analysis of gene by environmental interaction. However, it was so far, the first study to explore the interaction between vitamin D status and *PPARD*-rs6902123, and provided the suggestive evidence of interaction. Given the epidemic of vitamin D deficiency in the world [25], it is worthy of conducting more studies with large sample size to draw a definite conclusion.

In conclusion, we have reported for the first time that *PPARD*-rs6902123 C allele was associated with higher fasting glucose, HbA1c, and increased risk of type 2 diabetes and combined IFG/type 2 diabetes in a population-based Chinese Han sample. Moreover, we provided the suggestive evidence of interaction between 25(OH)D levels and *PPARD*-rs6902123 on HbA1c, indicating a possible crosstalk between PPAR-δ and vitamin D pathway.

## Supporting Information

Figure S1 Linkage disequilibrium among the eight SNPs in *PPARD* in our sample. The different shades and the figures stood for r^2^.
(TIF)

Table S1 Tag SNPs for PPARD gene region and alleles captured.
(DOC)

Table S2 Associations of variants in *PPARD* with type 2 diabetes and combined phenotype of type 2 diabetes and impaired fasting glucose in Chinese Hans.
(DOC)

Table S3 Associations with type 2 diabetes related quantitative traits in 2,943 Chinese Hans.
(DOC)
