# Supplementary figures and images for: Associations of Type 2 Diabetes with Common Variants in PPARD and the Modifying Effect of Vitamin D among Middle-Aged and Elderly Chinese
Source: PLoS One. 2012 Apr 11;7(4):e34895. doi: 10.1371/journal.pone.0034895 (PMC3324546; doi:10.1371/journal.pone.0034895)

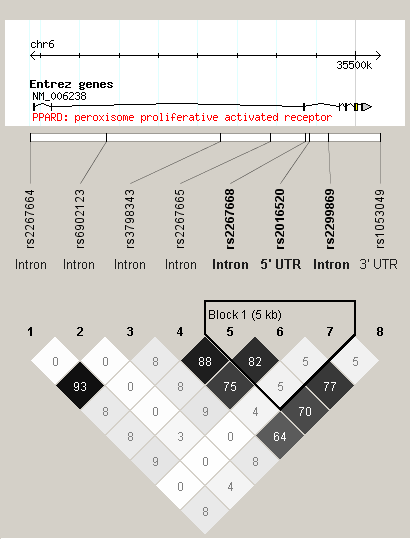

Supplement: Figure S1 — Linkage disequilibrium among the eight SNPs in PPARD in our sample. The different shades and the figures stood for r2. (TIF) [file pone.0034895.s001.tif]
