# Supplementary material for: Associations of Type 2 Diabetes with Common Variants in PPARD and the Modifying Effect of Vitamin D among Middle-Aged and Elderly Chinese
Source: PLoS One. 2012 Apr 11;7(4):e34895. doi: 10.1371/journal.pone.0034895 (PMC3324546; doi:10.1371/journal.pone.0034895)
Supplement: Table S1 — Tag SNPs for PPARD gene region and alleles captured. (DOC) [file pone.0034895.s002.doc]

**Table S1** Tag SNPs for PPARD gene region and alleles captured

| Tag SNPs | Alleles Captured |
| --- | --- |
| rs9658056 | rs9658056 |
| rs2267664 | rs2267664 |
| rs6902123 | rs6457816, rs9658100, rs7739752, rs9470007, rs6902123, rs9470001, rs6901410, rs6922548, rs9296148, rs6919334 |
| rs3798343 | rs3798343, rs12173582 |
| rs2267665 | rs9470015, rs9380506, rs2267665 |
| rs2267668 | rs9462082, rs760783, rs2267668, rs2267669, rs2076166, rs2076169 |
| rs2016520 | rs1883322, rs2076167, rs2267666, rs1040436, rs2267667, rs2016520, rs2038068 |
| rs2299869 | rs2299869 |
| rs1053049 | rs1053049, rs3734254 |
