# Supplementary material for: Associations of Type 2 Diabetes with Common Variants in PPARD and the Modifying Effect of Vitamin D among Middle-Aged and Elderly Chinese
Source: PLoS One. 2012 Apr 11;7(4):e34895. doi: 10.1371/journal.pone.0034895 (PMC3324546; doi:10.1371/journal.pone.0034895)
Supplement: Table S2 — Associations of variants in PPARD with type 2 diabetes and combined phenotype of type 2 diabetes and impaired fasting glucose in Chinese Hans. (DOC) [file pone.0034895.s003.doc]

**Table S2** Associations of variants in *PPARD* with type 2 diabetes and combined phenotype of type 2 diabetes and impaired fasting glucose in Chinese Hans

| SNP ID | Effect / Non-effect allele | *P*_HW_ | HapMap-CHB | HapMap-CEU | T2DM vs Normal | | | | (T2DM + IFG) vs Normal | | | |
| --- | --- | --- | --- | --- | --- | --- | --- | --- | --- | --- | --- | --- |
|  |  |  |  |  | Effect-allele frequency | | OR (95%CI) | *P* ^a^ | Effect-allele frequency | | OR (95%CI) | *P* ^a^ |
|  |  |  |  |  | Case  n=424 | Control  n=1908 |  |  | Case  n=1302 | Control  n=1908 |  |  |
| rs2267665 | [A / G] |  | 0.22 | 0.18 |  |  |  |  |  |  |  |  |
| Beijing |  | 0.9464 |  |  | 0.21 | 0.23 | 0.91 (0.71-1.17) | 0.4733 | 0.24 | 0.23 | 1.10 (0.93-1.31) | 0.2801 |
| Shanghai |  | 0.1485 |  |  | 0.25 | 0.24 | 1.13 (0.84-1.52) | 0.408 | 0.25 | 0.24 | 1.12 (0.93-1.36) | 0.2211 |
| Combined ^b^ |  |  |  |  |  |  | 1.00 (0.82-1.21) | 0.9709 |  |  | 1.11 (0.98-1.26) | 0.0988 |
| *P* for heterogeneity |  |  |  |  |  |  |  | 0.2772 |  |  |  | 0.8895 |
| rs2267668 | [G / A] |  | 0.21 | 0.18 |  |  |  |  |  |  |  |  |
| Beijing |  | 0.1125 |  |  | 0.21 | 0.23 | 0.9 (0.71-1.16) | 0.4282 | 0.24 | 0.23 | 1.06 (0.89-1.26) | 0.5422 |
| Shanghai |  | 0.1507 |  |  | 0.24 | 0.24 | 1.03 (0.77-1.4) | 0.8259 | 0.25 | 0.24 | 1.07 (0.88-1.29) | 0.5126 |
| Combined ^b^ |  |  |  |  |  |  | 0.96 (0.79-1.16) | 0.6581 |  |  | 1.06 (0.93-1.21) | 0.3601 |
| *P* for heterogeneity |  |  |  |  |  |  |  | 0.4952 |  |  |  | 0.963 |
| rs2016520 | [C / T] |  | 0.27 | 0.22 |  |  |  |  |  |  |  |  |
| Beijing |  | 0.2678 |  |  | 0.24 | 0.25 | 0.94 (0.74-1.19) | 0.5999 | 0.28 | 0.25 | 1.09 (0.92-1.28) | 0.3384 |
| Shanghai |  | 0.1246 |  |  | 0.29 | 0.27 | 1.2 (0.9-1.59) | 0.2088 | 0.29 | 0.27 | 1.16 (0.97-1.39) | 0.1009 |
| Combined ^b^ |  |  |  |  |  |  | 1.04 (0.86-1.24) | 0.6979 |  |  | 1.12 (0.99-1.26) | 0.0694 |
| *P* for heterogeneity |  |  |  |  |  |  |  | 0.1993 |  |  |  | 0.5869 |
| rs1053049 | [C / T] |  | 0.26 | 0.25 |  |  |  |  |  |  |  |  |
| Beijing |  | 0.1194 |  |  | 0.26 | 0.25 | 1.05 (0.83-1.34) | 0.6722 | 0.27 | 0.25 | 1.14 (0.96-1.35) | 0.1389 |
| Shanghai |  | 0.154 |  |  | 0.26 | 0.26 | 1.12 (0.83-1.5) | 0.4583 | 0.26 | 0.26 | 1.07 (0.89-1.3) | 0.4764 |
| Combined ^b^ |  |  |  |  |  |  | 1.08 (0.90-1.30) | 0.4267 |  |  | 1.11 (0.98-1.26) | 0.1068 |
| *P* for heterogeneity |  |  |  |  |  |  |  | 0.7715 |  |  |  | 0.6626 |
| rs3798343 | [G / C] |  | 0.27 | 0.02 |  |  |  |  |  |  |  |  |
| Beijing |  | 0.8286 |  |  | 0.27 | 0.28 | 0.94 (0.75-1.18) | 0.6131 | 0.28 | 0.28 | 0.97 (0.83-1.14) | 0.7221 |
| Shanghai |  | 0.5591 |  |  | 0.37 | 0.33 | 1.18 (0.92-1.52) | 0.1996 | 0.33 | 0.33 | 1.02 (0.87-1.21) | 0.7836 |
| Combined ^b^ |  |  |  |  |  |  | 1.04 (0.88-1.23) | 0.6278 |  |  | 1.0 (0.89-1.12) | 0.9724 |
| *P* for heterogeneity |  |  |  |  |  |  |  | 0.1849 |  |  |  | 0.6479 |
| rs2299869 | [T / C] |  | 0.15 | 0 |  |  |  |  |  |  |  |  |
| Beijing |  | 0.4431 |  |  | 0.20 | 0.16 | 1.27 (0.98-1.65) | 0.0668 | 0.16 | 0.16 | 0.97 (0.80-1.17) | 0.7258 |
| Shanghai |  | 0.7208 |  |  | 0.14 | 0.13 | 0.95 (0.66-1.36) | 0.7761 | 0.15 | 0.13 | 1.13 (0.90-1.42) | 0.2893 |
| Combined ^b^ |  |  |  |  |  |  | 1.15 (0.93-1.42) | 0.1951 |  |  | 1.03 (0.89-1.19) | 0.6795 |
| *P* for heterogeneity |  |  |  |  |  |  |  | 0.1955 |  |  |  | 0.3039 |
| rs2267664 | [A / G] |  | 0.23 | 0.03 |  |  |  |  |  |  |  |  |
| Beijing |  | 0.3308 |  |  | 0.26 | 0.28 | 0.94 (0.75-1.17) | 0.5553 | 0.27 | 0.28 | 0.98 (0.83-1.14) | 0.7652 |
| Shanghai |  | 0.6401 |  |  | 0.36 | 0.32 | 1.17 (0.91-1.51) | 0.2236 | 0.33 | 0.32 | 1.04 (0.88-1.23) | 0.6649 |
| Combined ^b^ |  |  |  |  |  |  | 1.03 (0.87-1.22) | 0.7049 |  |  | 1.00 (0.89-1.13) | 0.9435 |
| *P* for heterogeneity |  |  |  |  |  |  |  | 0.1921 |  |  |  | 0.5678 |

1. The *P* values were adjusted for age, sex, and BMI.
2. Fixed-effect model was used in the meta-analysis.
