# Supplementary material for: Associations of Type 2 Diabetes with Common Variants in PPARD and the Modifying Effect of Vitamin D among Middle-Aged and Elderly Chinese
Source: PLoS One. 2012 Apr 11;7(4):e34895. doi: 10.1371/journal.pone.0034895 (PMC3324546; doi:10.1371/journal.pone.0034895)
Supplement: Table S3 — Associations with type 2 diabetes related quantitative traits in 2,943 Chinese Hans. (DOC) [file pone.0034895.s004.doc]

**Table S3** Associations with type 2 diabetes related quantitative traits in 2,943 Chinese Hans

| SNP ID | Glucose (mmol/L) ^a^ | | HbA1c (%)^a^ | | Insulin (mmol/L) ^a b^ | | HOMA-B (%) ^a^ | | HOMA-IR (%) ^a b^ | |
| --- | --- | --- | --- | --- | --- | --- | --- | --- | --- | --- |
|  | *β* (SE) | *P* | *β* (SE) | *P* | *β* (SE) | *P* | *β* (SE) | *P* | *β* (SE) | *P* |
| rs2267665 |  |  |  |  |  |  |  |  |  |  |
| Beijing | -0.060 (0.064) | 0.3459 | -0.073 (0.041) | 0.0746 | 0.025 (0.023) | 0.2739 | -0.269 (1.829) | 0.8832 | 0.008 (0.021) | 0.717 |
| Shanghai | 0.043 (0.044) | 0.3293 | 0.010 (0.030) | 0.7482 | 0.027 (0.020) | 0.1854 | 1.397 (1.909) | 0.4644 | 0.026 (0.020) | 0.1986 |
| Combined ^c^ | 0.010 (0.036) | 0.7839 | -0.019 (0.024) | 0.434 | 0.026 (0.015) | 0.0833 | 0.528 (1.321) | 0.6891 | 0.017 (0.014) | 0.2286 |
| *P* for heterogeneity |  | 0.1848 |  | 0.1023 |  | 0.9477 |  | 0.5286 |  | 0.5348 |
| rs2267668 |  |  |  |  |  |  |  |  |  |  |
| Beijing | -0.052 (0.065) | 0.4266 | -0.057 (0.042) | 0.1742 | 0.017 (0.023) | 0.4524 | 0.682 (1.866) | 0.7149 | 0.010 (0.021) | 0.6515 |
| Shanghai | 0.017 (0.043) | 0.7012 | 0.013 (0.029) | 0.6512 | 0.010 (0.020) | 0.6035 | 1.176 (1.902) | 0.5365 | 0.009 (0.020) | 0.6688 |
| Combined ^c^ | -0.004 (0.036) | 0.9111 | -0.010 (0.024) | 0.6875 | 0.013 (0.015) | 0.3885 | 0.924 (1.332) | 0.4877 | 0.009 (0.014) | 0.5129 |
| *P* for heterogeneity |  | 0.3760 |  | 0.1702 |  | 0.8184 |  | 0.8529 |  | 0.9725 |
| rs2016520 |  |  |  |  |  |  |  |  |  |  |
| Beijing | -0.005 (0.062) | 0.9407 | -0.037 (0.040) | 0.3597 | 0.021 (0.022) | 0.3288 | 0.006 (1.779) | 0.9975 | 0.016 (0.020) | 0.4223 |
| Shanghai | 0.055 (0.042) | 0.1909 | 0.042 (0.028) | 0.1359 | 0.009 (0.020) | 0.6523 | -0.381 (1.839) | 0.8359 | 0.008 (0.019) | 0.6617 |
| Combined ^c^ | 0.036 (0.035) | 0.2988 | 0.016 (0.023) | 0.4849 | 0.014 (0.015) | 0.3295 | -0.181 (1.279) | 0.8874 | 0.012 (0.014) | 0.3919 |
| *P* for heterogeneity |  | 0.4230 |  | 0.1057 |  | 0.6865 |  | 0.8798 |  | 0.7718 |
| rs1053049 |  |  |  |  |  |  |  |  |  |  |
| Beijing | 0.011 (0.064) | 0.8659 | -0.021 (0.041) | 0.6047 | 0.017 (0.023) | 0.4629 | -0.220 (1.840) | 0.9046 | 0.012 (0.021) | 0.5826 |
| Shanghai | 0.044 (0.044) | 0.3143 | 0.038 (0.029) | 0.1909 | 0.003 (0.020) | 0.8975 | -0.088 (1.907) | 0.9632 | 0.002 (0.020) | 0.9287 |
| Combined ^c^ | 0.033 (0.036) | 0.3568 | 0.018 (0.024) | 0.4389 | 0.009 (0.015) | 0.5497 | -0.156 (1.324) | 0.906 | 0.007 (0.014) | 0.6409 |
| *P* for heterogeneity |  | 0.6709 |  | 0.2401 |  | 0.646 |  | 0.9603 |  | 0.7302 |
| rs3798343 |  |  |  |  |  |  |  |  |  |  |
| Beijing | -0.076 (0.060) | 0.2055 | -0.021 (0.039) | 0.5905 | -0.014 (0.021) | 0.5025 | 2.138 (1.724) | 0.215 | -0.016 (0.020) | 0.4381 |
| Shanghai | -0.033 (0.039) | 0.3964 | -0.011 (0.026) | 0.6812 | -0.016 (0.018) | 0.3817 | -0.547 (1.710) | 0.7493 | -0.018 (0.018) | 0.3022 |
| Combined ^c^ | -0.046 (0.033) | 0.1616 | -0.014 (0.022) | 0.5152 | -0.015 (0.014) | 0.2675 | 0.785 (1.214) | 0.5181 | -0.017 (0.013) | 0.2011 |
| *P* for heterogeneity |  | 0.5479 |  | 0.8311 |  | 0.9423 |  | 0.2688 |  | 0.9408 |
| rs2299869 |  |  |  |  |  |  |  |  |  |  |
| Beijing | 0.079 (0.075) | 0.2884 | 0.068 (0.048) | 0.1617 | -0.040 (0.026) | 0.127 | -3.054 (2.118) | 0.1496 | -0.040 (0.025) | 0.1018 |
| Shanghai | 0.054 (0.054) | 0.3140 | 0.025 (0.036) | 0.4862 | -0.002 (0.025) | 0.9296 | -2.604 (2.350) | 0.268 | 0.004 (0.025) | 0.8615 |
| Combined ^c^ | 0.063 (0.044) | 0.1536 | 0.040 (0.029) | 0.1599 | -0.020 (0.018) | 0.2610 | -2.852 (1.573) | 0.0698 | -0.018 (0.018) | 0.3086 |
| *P* for heterogeneity |  | 0.7868 |  | 0.4736 |  | 0.2921 |  | 0.8869 |  | 0.2133 |
| rs2267664 |  |  |  |  |  |  |  |  |  |  |
| Beijing | -0.051 (0.060) | 0.3921 | -0.016 (0.039) | 0.6789 | -0.002 (0.021) | 0.9075 | 2.041 (1.713) | 0.2336 | -0.006 (0.020) | 0.7772 |
| Shanghai | -0.028 (0.039) | 0.4706 | -0.014 (0.027) | 0.6064 | -0.015 (0.018) | 0.4219 | -0.647 (1.720) | 0.7067 | -0.017 (0.018) | 0.3430 |
| Combined ^c^ | -0.035 (0.033) | 0.2868 | -0.015 (0.022) | 0.5094 | -0.009 (0.014) | 0.4873 | 0.702 (1.214) | 0.5627 | -0.012 (0.013) | 0.3667 |
| *P* for heterogeneity |  | 0.7479 |  | 0.9664 |  | 0.6383 |  | 0.2682 |  | 0.6827 |

a. The *P* values were adjusted for age, sex and BMI.

b. Log-transformed before analyses.

c. Fixed-effect model was used in the meta-analysis.
